# T-Cell Lymphopenia Detected by Newborn Screening in Two Siblings with an Xq13.1 Duplication

**DOI:** 10.3389/fped.2017.00156

**Published:** 2017-07-18

**Authors:** Xavier Rios, Ivan K. Chinn, Jordan S. Orange, Celine I. Hanson, Nicholas L. Rider

**Affiliations:** ^1^Center for Human Immunobiology, Baylor College of Medicine, Texas Children’s Hospital, Houston, TX, United States

**Keywords:** severe combined immunodeficiency, lymphopenia, T-cell receptor excision circles, primary immunodeficiency, idiopathic T-cell lymphopenia, Xq13.1

## Abstract

Newborn screening for severe combined immunodeficiency has proven successful in identifying infants with T-cell deficiencies before they become severely ill. Additionally, the newborn screen can detect subtle early phenotypes that may become severe later in life. We present the case of siblings with features suggestive of T-cell lymphopenia identified as having low T-cell receptor excision circles counts by newborn screening. Expanded immune testing showed robust lymphocyte mitogen and antigen responses with normal vaccine responses and immunoglobulin levels for both boys over time. Genetic analysis revealed an Xq13.1 duplication in each child not found in the mother. The variant is downstream of the IL2RG gene with potential regulatory significance, suggesting a mechanism for the T-cell lymphopenia. The newborn screen provided these patients heightened surveillance and patient-specific management, including delayed live vaccines and *Pneumocystis jiroveci* pneumonia prophylaxis. Fortunately, the brothers have not suffered invasive or opportunistic infections and are well at ages 3 and 4 years. In this report, we illustrate the challenges of managing seemingly asymptomatic immunodeficient patients without a definitive genetic diagnosis and show how unbiased genetic analysis can expand understanding about primary immunodeficiency phenotypes.

## Introduction

The newborn screen T-cell receptor excision circles (TRECs) assay successfully identifies patients with significant T-cell lymphopenia before they become severely ill. As a marker for recent thymic emigrants, low numbers of TRECs serve as a proxy for T-cell lymphopenia, a key characteristic of many primary immunodeficiencies, including severe combined immunodeficiency (SCID) (1). However, the sensitivity of the TREC screen allows for detection of milder T-cell deficiency phenotypes as well. These immunodeficiencies have variability in age of onset, clinical manifestations, and immunological parameters (2). One of these intermediate phenotypes is “leaky” SCID, defined by low T-cell counts (300–1,500 cells/μL) and reduced proliferation to mitogens, most notably phytohemagglutinin. Idiopathic T-cell lymphopenia might represent a milder form of a SCID phenotype spectrum, with low T-cell counts but normal proliferation to mitogens (3). For example, mutations in the *RAG1* gene can cause a broad range of immunodeficiency presentations (4, 5), including a case of idiopathic T-cell lymphopenia seen in a 5-year-old girl with varicella pneumonia (6). Therefore, idiopathic T-cell lymphopenia might be caused by milder genetic defects in SCID-causing genes.

### Cases

**Figure 1:**
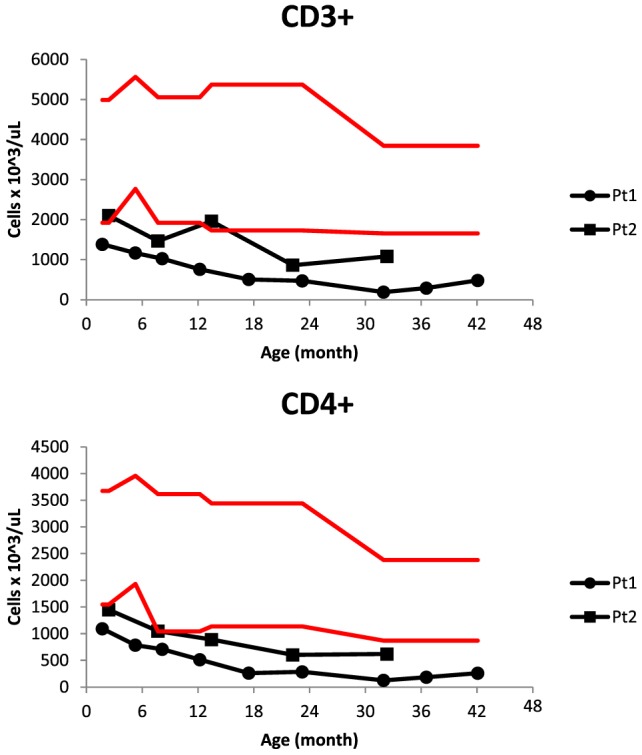
CD3^+^ and CD4^+^ cell counts in progressive lymphopenia of two siblings with low T-cell receptor excision circles on newborn screening. Red lines delimit the normal range for age.

Patient 1 was a male born at 37 weeks of gestational age to an African-American mother with gestational diabetes controlled by insulin. He underwent evaluation at 7 weeks of age after his newborn screens from birth and 2 weeks revealed TREC of 80 and 0 copies/mL, respectively. There was no family history of early infant deaths or consanguinity. Clinical findings were notable for mild facial dysmorphism, low set ears, and transverse phalangeal creases. The immunologic evaluation revealed T-cell subset abnormalities: decreased CD4^+^ and CD8^+^ cell counts with conserved CD4:CD8 ratio and elevated CD20^+^ and HLA-DR^+^ cells (Table 1). Otherwise, he had normal immunoglobulin levels, normal NK cell counts, normal lymphocyte proliferation to antigens and mitogens, and appropriate antibody responses to diphtheria, tetanus, and conjugated *Streptococcus pneumoniae* vaccination. Live vaccines were delayed initially until repeat testing showed adequate lymphocyte proliferation to antigens and mitogens. He tolerated varicella and measles, mumps, and rubella vaccines. At 17 months, his CD4^+^ T-cell counts dropped below 300 cells/μL (Figure 1), and he started Bactrim for *Pneumocystis jiroveci* pneumonia (PJP) prophylaxis.

**Table 1:** Immunologic laboratory values for siblings with idiopathic T-cell lymphopenia.

Lab value (units)	Patient 1	Patient 2
IgG (mg/dL)	514 (207–904)	597 (207–904)
IgA (mg/dL)	15 (10–45)	29 (10–50)
IgM (mg/dL)	40 (15–96)	52 (18–80)
IgE (IU/mL)	5.6 (1.5–52.3)	11.4 (1.5–52.3)
Diphtheria toxin IgG antibody (IU/mL)	6.4 (>0.1)	ND
Tetanus toxoid IgG antibody (IU/mL)	0.8	ND
*S. pneumoniae* antibody Type 1 (μg/mL)	>33.02 (>1)	ND
*S. pneumoniae* antibody Type 3 (μg/mL)	2.28 (>1)	ND
*S. pneumoniae* antibody Type 4 (μg/mL)	>11.97 (>1)	ND
*S. pneumoniae* antibody Type 5 (μg/mL)	>42.77 (>1)	ND
*S. pneumoniae* antibody Type 6B (μg/mL)	3.21 (>1)	ND
*S. pneumoniae* antibody Type 7F (μg/mL)	10.06 (>1)	ND
*S. pneumoniae* antibody Type 8 (μg/mL)	0.15 (>1)	ND
*S. pneumoniae* antibody Type 9N (μg/mL)	3.77 (>1)	ND
*S. pneumoniae* antibody Type 9V (μg/mL)	13.73 (>1)	ND
*S. pneumoniae* antibody Type 12F (μg/mL)	0.26 (>1)	ND
*S. pneumoniae* antibody Type 14 (μg/mL)	13.58 (>1)	ND
*S. pneumoniae* antibody Type 18C (μg/mL)	>47.50 (>1)	ND
*S. pneumoniae* antibody Type 19F (μg/mL)	>59.27 (>1)	ND
*S. pneumoniae* antibody Type 23F (μg/mL)	>18.35 (>1)	ND
PHA 10 µg/mL (163,507–415,087 cpm)	275,728	101,120
1.0 µg/mL (35,494–225,107 cpm)	51,457	15,990
ConA 50 µg/mL (80,718–286,866 cpm)	207,275	38,039
5.0 µg/mL (28,998–108,585 cpm)	84,863	33,107
PWM 100 ng/mL (37,006–157,955 cpm)	76,678	50,290
10 ng/mL (24,369–94,311 cpm)	43,854	11,582
Unstimulated (215–1,161 cpm)	445	236
Candida antigen (>2,000 cpm)	10,267	18,283
Diphtheria antigen (>2,000 cpm)	15,633	18,642
Tetanus antigen (>2,000 cpm)	2,336	12,370
CD3^+^ (1,920–4,991 10^3^ cell/UL)	1,382	2,101
CD3^+^CD4^+^ (1,546–3,673 10^3^ cell/UL)	1,092	1,447
CD3^+^CD8^+^ (359–1,489 10^3^ cell/UL)	276	600
CD19^+^ (256–1,579 10^3^ cell/UL)	2,765	3,242
CD3^−^CD56^+^CD16^+^ (116–783 10^3^ cell/UL)	415	480
HLA-DR^+^ (59–457 10^3^ cell/UL)	3,087	3,722
CD3^+^HLA-DR^+^ (0–250 10^3^ cell/UL)	92	180
CD20^+^ (59–457 10^3^ cell/UL)	2,949	3,302
CD2^+^ (1,009–1,936 10^3^ cell/UL)	1,382	38
CD56^+^ (137–478 10^3^ cell/UL)	323	360
CD8^+^CD56^+^ (30–200 10^3^ cell/UL)	138	120
CD4^+^CD45RA^+^ (134–969 10^3^ cell/UL)	783	1,141
CD4^+^CD45RO^+^ (301–919 10^3^ cell/UL)	230	360
CD19^+^CD27^+^ (19–131 10^3^ cell/UL)	23	30
HIV 1 DNA PCR	Not detected	ND

*Age-appropriate normal ranges in parenthesis*.

*ND, not done*.

Soon after birth, he became hypoxemic during feeds and a swallow study showed severe gastroesophageal reflux disease (GERD). The reflux likely led to failure to thrive with dysphagia and recurrent emesis that resolved at 23 months. He was hospitalized at 2 months of age to expedite workup for his positive TREC screen result. During this hospilatization, he developed a fever to 39.3 F, with nasal washings PCR positive for rhinovirus and parainfluenza. Subsequently, he has suffered occasional upper respiratory infections, acute otitis media approximately two to four times per year, two episodes of hand, foot, and mouth disease, and two episodes of mild pneumonia treated as an outpatient. He has history of mild developmentally delayed, walking at 14 months and speaking 10–15 words at 24 months. Reportedly, the two older siblings and his Hispanic father have developmental delay and intellectual disability.

Patient 2 was a male born at 35 weeks and 6 days gestational age with a pregnancy complicated by gestational diabetes. His newborn screening also revealed very low TRECs. In the first weeks of life, he was hospitalized for hypoglycemia, likely from gestational diabetes and then bronchiolitis, recovering uneventfully. Other infectious complications included several episodes of otitis media, hand, foot, and mouth disease, and influenza; like his brother, he recovered. Unlike his older brother, he did not have clinical GERD, failure to thrive, or developmental delay. His immunologic evaluation also revealed normal immunoglobulin levels, normal NK cell counts, normal lymphocyte responses to mitogens and antigens, and decreased T-cell counts, albeit less severe than his brother (Table 1; Figure 1).

### Genetic Analysis

**Figure 2:**
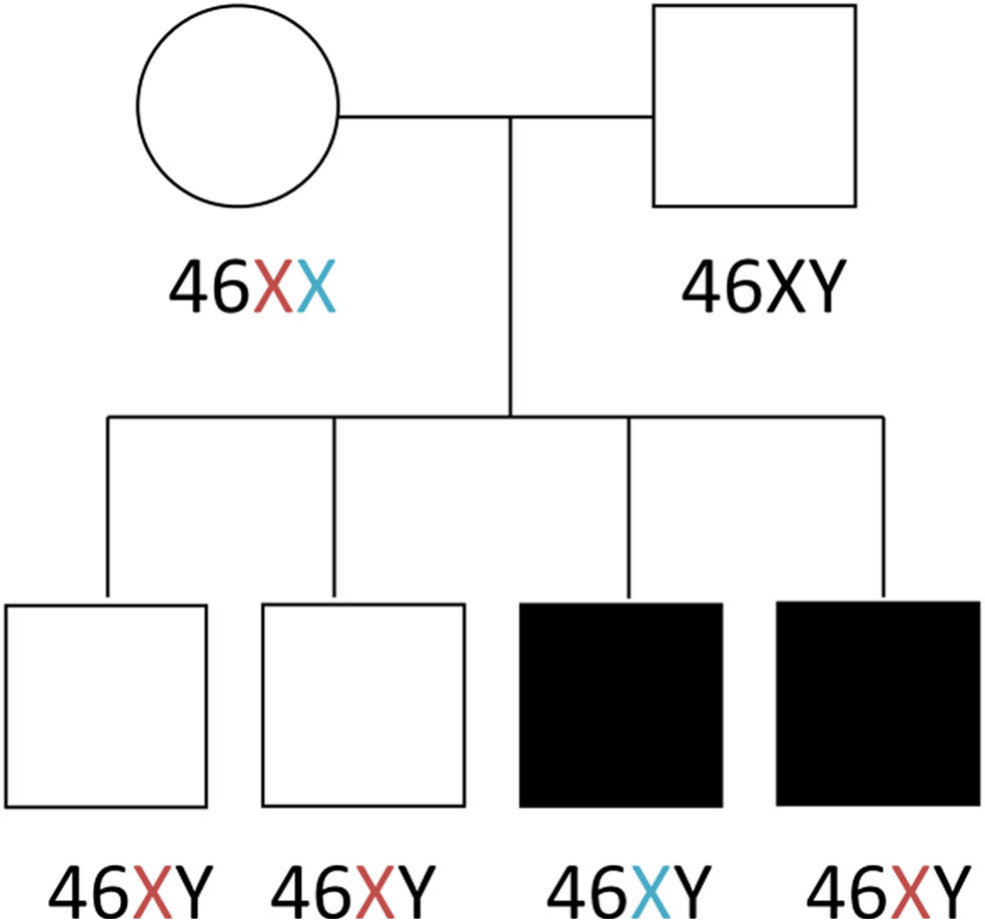
Family pedigree.

Genetic workup *via* chromosomal microarray revealed a 1–3 kb Xq13.1 duplication covering exons 25–28 of the *MED12* gene. The duplication was present in both affected siblings, but absent in two healthy siblings and both parents (Figure 2). Identification of the Xq13.1 duplication was followed by whole exome sequencing of all family members. A list of the genetic variants that segregate only with the affected siblings is provided (Table 2). These variants are not in genes known to be involved in primary immunodeficiencies. Exome sequencing revealed that the two siblings inherited different X chromosomes from their mother, which argues against a maternal balanced translocation. Gonadal mosaicism may explain how the two siblings can have a duplication not seen in their parents, a mechanism reported in cases of Wiskott–Aldrich syndrome (7) and X-linked SCID (8, 9).

**Table 2:** Results of exome sequencing.

Gene	Genomic coordinates	Zygosity	ExAC allelic frequency	Gene function
CTAGE15P	chr7:143269281_A > C	Hom	0	Pseudogene, cutaneous T-cell lymphoma-associated antigen family
TBC1D8B	chrX:106082584_T > G	Hom	0.0006	May act as a GTPase-activating protein for Rab family protein(s)
NAP1L3	chrX:92926826_T > C	Hom	0.0006	Intron-less gene that encodes a member of the nucleosome assembly protein family. Closely linked to a region of genes responsible for several X-linked mental retardation syndromes
PPARGC1B	chr5:149200068_G > A	Het	0.00002	The protein encoded by this gene stimulates the activity of several transcription factors and nuclear receptors, including estrogen receptor alpha, nuclear respiratory factor 1, and glucocorticoid receptor
OR1S2^a^	chr11:57970695_C > G	Het (maternal)	0.00003	Odorant receptor
OR1S2^a^	chr11:57971546_A > C	Het (paternal)	0.003	Odorant receptor

*The six gene variants found only present in affected siblings and not unaffected family members*.

*^a^Compound heterozygous*.

### Consent

Informed consent for research studies for the patients and parents, including genetic testing, were obtained through BCM Institutional Review Board approved protocols. Both parents provided informed consent for participation.

## Background

T-cell receptor excision circles screening identifies patients born with low T-cell counts. The test has been successful not only at detecting SCID but also other conditions such as idiopathic T-cell lymphopenia. The frequency of idiopathic T-cell lymphopenia detected by TRECs screening is estimated at 1 per 250,000 births (3). This condition is a diagnosis of exclusion and needs to be distinguished from other causes of newborn T-cell lymphopenia such as prematurity or neonatal illness. Longitudinal testing will show recovery of cell counts in those conditions, while idiopathic T-cell lymphopenia will remain with low T-cell counts. An analogous condition in adults, idiopathic CD4^+^ lymphocytopenia, was described in a prospective cohort study involving 39 patients who were diagnosed *via* an incidental laboratory finding or an unexplained opportunistic infection (10). Although it was termed CD4^+^ lymphopenia, about 40% of these patients had both low CD4^+^ and CD8^+^ cell counts, which was associated with higher risk of infections and mortality. A literature review of 259 reported idiopathic CD4^+^ lymphopenia cases found that at diagnosis the mean age was 40.7 years, 62% of cases were male, 87.6% had one or more opportunistic infection, 14.2% had an autoimmune disease, and 18.1% had a malignancy (11). Several T-cell abnormalities have been detected in patients with idiopathic CD4^+^ lymphopenia, including overexpression of Fas leading to increased apoptosis (12) and decreased surface expression of CXCR4 (13).

## Discussion

**Figure 3:**
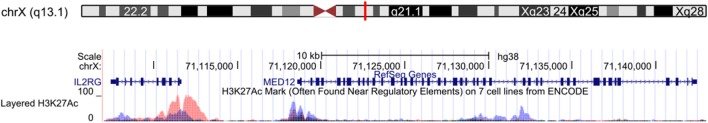
Xq13.1 duplication region shared by affected siblings. The red box shows exons 25–28 of the MED12 gene, which were duplicated in the two affected patients with idiopathic T-cell lymphopenia. This region contains H3K2AC histone marks from the Encyclopedia of DNA Elements. UCSC Genome Browser GRCh38/hg38 assembly (16).

We report two brothers with low TREC levels and idiopathic T-cell lymphopenia. The T-cell lymphopenia in our patients has been persistent and progressive, arguing against secondary causes such as prematurity. Even though Patient 1 had a slightly sicker course during early infancy, this was likely due to his reflux and failure to thrive. They both have normal *in vitro* T-cell function and ability to combat viral infections, arguing against a “leaky” SCID diagnosis. In addition, both of them have an Xq13.1 duplication region is downstream of the IL2RG gene, the causal gene of the most common form of SCID, X-linked SCID (14). Based on the Encyclopedia of DNA Elements data, this region might be of regulatory significance (Figure 3). It is tempting to hypothesize that copy number changes of this potential regulatory region could lead to dysregulation of the IL2RG gene and impaired homeostatic maintenance of T-cell levels. However, our patients have normal NK and B cell counts, which is not expected in X-linked SCID. One explanation for this could be pleiotropy of a mutation involving a regulatory region, leading to differential cell type phenotypes. Alternatively, the Xq13.1 duplication might have inserted in an immunologically relevant location, such as gene necessary for T-cell development or survival. Such a rearrangement could cause idiopathic T-cell lymphopenia, as has been previously reported in other cases (6, 15). Finally, Xq13.1 duplication could be an incidental finding unrelated to the T cell lymphopenia. Further studies are needed to further elucidate the genetic basis of our patient’s phenotype.

One advantage of early T-cell lymphopenia detection was that heightened surveillance lead to patient-specific medical management. For example, live vaccines were delayed until longitudinal testing showed normal T -cell responses to antigens and mitogens and appropriate responses to non-live vaccines. Furthermore, Patient 1 was started on PJP prophylaxis once his CD4^+^ cell counts dropped below 300 cells/μL, while Patient 2 has not required this. It is unclear why the older sibling has lower counts, although his initial failure to thrive might have contributed to it. Alternatively, their underlying genetic disorder might exhibit variable phenotypic penetrance.

The cases reported here illustrate the challenge of managing asymptomatic patients with idiopathic T-cell lymphopenia detected by the TREC screen. The non “full SCID” immunodeficiencies detected by the TREC assay have a variable age of onset, clinical manifestations, and immunological parameters. SCID and “leaky” SCID display reduced mitogen proliferation studies and significant susceptibility to infection, justifying bone marrow transplantation. In idiopathic T-cell lymphopenia, it is unclear if the immune function will remain normal or deteriorate over time. Longitudinal tracking of these patients will be essential for understanding their phenotype. It may be discovered that some of the patients diagnosed during infancy with idiopathic T-cell lymphopenia represent the earliest presentation of the idiopathic CD4-cell lymphopenia described in adults.

It is critical to know if our patients’ defect is known or expected to worsen and evolve “leaky” SCID. A causal genetic diagnosis would be valuable because it can delineate a clinical course and pathway-specific management. However, whole exome sequencing did not detect any mutations in genes known to cause immunodeficiency, highlighting an advantage functional screens such as TREC have over genetic testing. It is likely that the subtler phenotype of idiopathic T-cell lymphopenia might be due to either polygenic interactions or alterations in regulatory regions not captured by exome sequencing. Whole genome sequencing may identify additional non-coding variants to explain the T-cell lymphopenia and help elucidate whether the findings of Xq13.1 duplications in these two boys inherited on differing X chromosomes is relevant to their immune findings. Complementing this technique with functional studies of the discovered genetic variants may lead to mechanistic insights that explain the T-cell lymphopenia. Such information could guide medical management; for example, if our patients have a regulatory defect leading to low IL-2 levels, replacement may promote homeostatic proliferation of lymphocytes (13, 17).

The goal of TREC screening is to identify patients with SCID whose lives can be saved with early bone marrow transplantation. The screen, however, is also revealing new intermediate immunodeficiency phenotypes with unknown natural history and for which clinical management is less clear. Further studying of these cases with advance genetic testing can lead to new insights in immunology that will further expand the utility of screening and improve outcomes in patients with primary immunodeficiencies.

## Author Contributions

XR studied cases and wrote manuscript, IC contributed with the genetic analysis and manuscript writing, JO contributed to clinical analysis and manuscript writing, CH contributed with identification and longitudinal assessment of patient, clinical analysis, and manuscript writing, NR contributed with clinical analysis and manuscript writing.

## Conflict of Interest Statement

The authors declare that the research was conducted in the absence of any commercial or financial relationships that could be construed as a potential conflict of interest.
